# Sarcopenia Does Not Affect Survival or Outcomes in Soft-Tissue Sarcoma

**DOI:** 10.1155/2015/146481

**Published:** 2015-12-01

**Authors:** Robert J. Wilson, Vignesh K. Alamanda, Katherine G. Hartley, Nathan W. Mesko, Jennifer L. Halpern, Herbert S. Schwartz, Ginger E. Holt

**Affiliations:** ^1^Department of Orthopaedics and Rehabilitation, Vanderbilt University Medical Center, Nashville, TN 37232, USA; ^2^Department of Orthopaedics, Carolinas Medical Center, Charlotte, NC 28203, USA; ^3^Department of Radiology and Radiological Sciences, Vanderbilt University Medical Center, Nashville, TN 37232, USA; ^4^Department of Orthopaedics, Cleveland Clinic, Cleveland, OH 44195, USA

## Abstract

*Background and Objective*. Sarcopenia is associated with decreased survival and increased complications in carcinoma patients. We hypothesized that sarcopenic soft-tissue sarcoma (STS) patients would have decreased survival, increased incidence of wound complications, and increased length of postresection hospital stay (LOS).* Methods*. A retrospective, single-center review of 137 patients treated surgically for STS was conducted. Sarcopenia was assessed by measuring the cross-sectional area of bilateral psoas muscles (total psoas muscle area, TPA) at the level of the third lumbar vertebrae on a pretreatment axial computed tomography scan. TPA was then adjusted for height (cm^2^/m^2^). The association between height-adjusted TPA and survival was assessed using Cox proportional hazard model. A logistical model was used to assess the association between height-adjusted TPA and wound complications. A linear model was used to assess the association between height-adjusted TPA and LOS.* Results*. Height-adjusted TPA was not an independent predictor of overall survival (*p* = 0.746). Patient age (*p* = 0.02) and tumor size (*p* = 0.009) and grade (*p* = 0.001) were independent predictors of overall survival. Height-adjusted TPA was not a predictor of increased hospital LOS (*p* = 0.66), greater incidence of postoperative infection (*p* = 0.56), or other wound complications (*p* = 0.14).* Conclusions*. Sarcopenia does not appear to impact overall survival, LOS, or wound complications in patients with STS.

## 1. Introduction

Sarcopenia, defined as the age-associated loss of skeletal muscle mass and function [1], has emerged as an important variable for predicting survival and complications for multiple malignancies. Sarcopenia has been shown to be an independent predictor of survival in patients with hepatocellular carcinoma [2], pancreatic adenocarcinoma [3], melanoma [4], and breast cancer [5]. Patients with sarcopenia have also been shown to be significantly more likely to have dose limiting toxicity from chemotherapeutic medications for renal cell carcinoma [6, 7], hepatocellular carcinoma [8], breast cancer [9], and thyroid cancer [10]. In addition, sarcopenia is associated with increased incidence of postoperative infection and length of hospital stay (LOS) in patients after colorectal cancer resections [11] and increased major complication rate and LOS in patients undergoing liver resection for colorectal cancer metastases [12].

The exact method of calculation of sarcopenia has been variable in the literature. However, calculating the area of skeletal muscle mass using computed tomography (CT) scans has become a commonly accepted modality for calculating sarcopenia and is considered by some to be the “gold standard” [13]. The use of the third or fourth lumbar vertebral level on an axial CT has become a common reference standard in multiple studies [3, 4, 11, 13] and a single cross-sectional slice for calculating muscle area has been shown to correlate strongly with whole body muscle mass [14, 15].

The numerical values for what constitutes sarcopenia are variable as well. Prado et al., in patients with gastrointestinal and pulmonary malignancies, established sex-specific cutoffs for sarcopenia: below 52.4 cm²/m² for men and 38.5 cm²/m² for women, respectively, taken from mortality-based statistical stratification [16]. These values have since become reference standards for defining sarcopenia in multiple subsequent studies of carcinoma patients [8, 9, 11]. However, two other studies have used different cutoff values (43.75 cm^2^/m^2^ for men and 41.10 cm^2^/m^2^ for women) based on overall survival in patients undergoing resection of colorectal liver metastases [2, 17]. Yet another study defined sarcopenia as a total psoas area (TPA: combined surface area of bilateral psoas muscles) of less than 500 mm^2^/m^2^ based on optimum stratification [12].

To our knowledge, no prior studies exist that evaluate the relationship of sarcopenia to overall survival and treatment complications in patients with extremity soft-tissue sarcomas (STS) undergoing surgical resection. There are no established values for what constitutes sarcopenia in patients with soft-tissue sarcomas. We hypothesized that patients with STS and sarcopenia, as manifest by decreased height-adjusted TPA, would have decreased overall survival, increased incidence of postoperative wound complications including infection, and increased postresection LOS.

## 2. Materials and Methods

**Figure 1:**
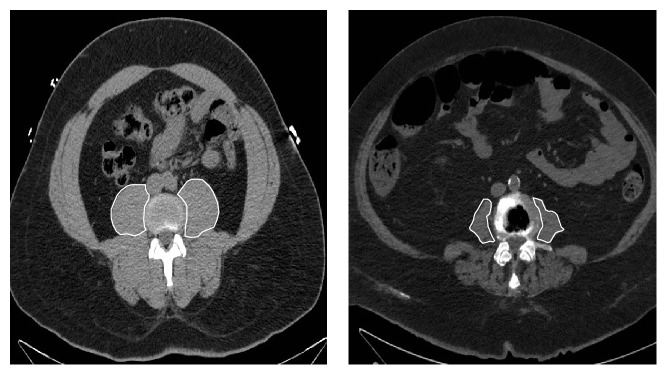
Example calculation method for TPA based on axial CT scan at L3 in two patients. Notice the difference in TPA between them.

A retrospective, single-center review of 137 patients treated surgically for extremity STS between 2000 and 2008 was conducted. Relevant oncologic data and body mass index (weight (kg)/height (m^2^)) were retrospectively extracted from our electronic medical record. Sarcopenia was assessed by manually measuring the cross-sectional area of the right and left psoas muscles (total psoas muscle area, TPA) on a single slice from a preoperative, staging CT scan at the level of the third lumbar vertebrae using Impax imaging software (version 6.3, Agfa Healthcare, Mortsel, Belgium), similar to the method outlined by Peng et al. [3]. TPA was then adjusted for height (cm^2^/m^2^). We chose this technique because it has been shown to be a valid method to calculate sarcopenia in the Peng et al. study and for the ease of manual measurement, given that only two muscle areas need to be traced out, as shown by the comparison scans of two patients in Figure 1.

In contrast, calculating the entire cross-sectional muscle area manually at the L3 level, unless aided by expensive computer software, is more time-consuming and potentially less reliable.

We used Wilcoxon rank sum test to compare continuous variables between two groups and Pearson chi-squared test to compare the categorical variables between groups. The association between height-adjusted TPA, age, gender, tumor grade, tumor depth, tumor size, surgical resection margin status, and overall survival was assessed using Cox proportional hazard model. A logistical model was used to assess the association between height-adjusted TPA and wound complications including infection with adjustment for age and gender. A wound infection was defined as requiring (1) oral or intravenous antibiotics, (2) in-clinic irrigation and debridement plus antibiotics, or (3) surgical irrigation and debridement plus antibiotics within 6 months of definitive sarcoma resection. Wound complications other than infection were defined as wound dehiscence requiring multiple gauze packing or negative pressure dressing changes or wound/flap necrosis requiring packing or debridement without need for antibiotics.

We applied a linear model with ordinary least squares to assess the association between height-adjusted TPA and length of stay with adjustment for age and gender. We did not establish gender-specific cutoffs to define sarcopenia as outlined elsewhere [16] as this is the first study to analyze sarcopenia in sarcoma patients. We instead linearly compared height-adjusted TPA with hospital LOS, wound infections, and wound complications as outlined above while controlling for gender. If sarcopenia, as manifested by decreased height-adjusted TPA, was found to be a significant predictor of poor outcomes on linear analysis, then risk stratification would be performed to establish appropriate cutoff values. A *p* value <0.05 was considered statistically significant. Statistical analyses were performed using open source R statistical software (version 3.0.2, Vienna, Austria) and SPSS (version 23, Armonk, NY). All statistical analyses were two-tailed. Our institution's internal review board gave approval for the study. No outside source of funding was used.

## 3. Results

Summary demographic, clinical, and outcome statistics are shown in Tables 1 and 2.

Height-adjusted TPA differed significantly between men and women (*p* < 0.001) (Table 2). Height-adjusted TPA was not an independent predictor of overall survival (*p* = 0.746), even after controlling for gender (*p* = 0.712), in Cox proportional hazard model as shown in Table 3. Similar to prior studies, both tumor size (*p* = 0.009) and grade (*p* = 0.001) were independent predictors of overall survival (Table 3). We also found increasing age (*p* = 0.02) to be an independent predictor of overall survival in Cox proportional hazard model. Tumor depth relative to investing fascia was not a significant predictor of survival (*p* = 0.337). Presence of a positive resection margin was not a significant predictor of survival as shown in Table 3 (*p* = 0.422).

Height-adjusted TPA was not a significant predictor of increased hospital LOS (*p* = 0.66), greater incidence of postoperative infection (*p* = 0.56), or incidence of other wound complications including dehiscence (*p* = 0.14), even after controlling for gender as shown in Table 4.

## 4. Discussion

This study is the first to analyze the influence of sarcopenia on survival and postoperative complications in patients with STS. Our study has several weaknesses. First, it is a single-center study with a relatively small number of patients and therefore the negative results could be due to the study being underpowered. However, multiple studies in the carcinoma literature have found sarcopenia to be a statistically significant predictor of outcomes, each with less patients enrolled than the 137 patients enrolled in this study [4, 7–9]. Second, the study is retrospective and does not contain any cancer-free control subjects. Third, our group of sarcomas was heterogeneous with regard to tumor grade, histologic subtype, and use of chemotherapy which may have influenced our results. Lastly, TPA was calculated based on manual measurements by authors Katherine G. Hartley and Vignesh K. Alamanda and may not be as precise as alternative methods such as using specially designed computer software.

We found no link between sarcopenia as manifest by height-adjusted TPA and overall survival, adverse events such as postoperative wound complications including infection, or increased hospital LOS. Using Cox proportional hazard model, we found that tumor grade and size were independent predictors of overall survival, similar to previous studies [18–20]. We also found that patient age was an independent predictor of overall survival, which has also been shown elsewhere [20].

The reasons our study did not find a link between sarcopenia, survival, LOS, and wound outcomes in STS are likely multifactorial. The average male and female height-adjusted TPA, shown in Table 2, were well above the 500 mm^2^/m^2^ TPA cutoff for sarcopenia used elsewhere [12]. Therefore the incidence, pathogenesis, and consequence of cancer cachexia and muscle wasting may be different in sarcomas than in carcinomas. However, we could find no studies in the literature looking at the clinical incidence or molecular and biochemical pathogenesis of muscle wasting and cachexia in soft-tissue sarcoma specifically. The predilection of most STS to metastasize to the lung hematogenously [21], in contradistinction to most carcinomas, is likely indicative of differential use of molecular signaling pathways and gene expression between the two cancer types [22].

In addition, 20% of the sarcomas in this study were superficial to the investing fascia, meaning direct structural influence on skeletal muscle was certainly limited and biochemical influence may be limited as well. The literature showing worse outcomes in carcinoma patients with sarcopenia includes multiple visceral abdominal tumors such as pancreatic, liver, and colorectal cancer which likely have a direct structural, as well as biochemical, effect on nutritional status that may be more significant than extremity sarcomas. For example, Lieffers et al. found that patients presenting with bowel obstruction from colorectal cancer were much more likely to have sarcopenia [11]. Our study also found no significant relationship between sarcopenia and postoperative wound complications, including infection. The principle study [11] to have shown a link between sarcopenia and “postoperative infections” also included postoperative pneumonia and urinary tract infections in addition to wound infections, which we did not.

The impact of sarcopenia on LOS has been variable with some carcinoma studies [11, 12] finding LOS increased in sarcopenic patients while another found no difference [3]. LOS on average in our study was 4 days, while the LOS in other studies with pancreaticoduodenectomies, liver resection, and bowel resections [3, 11, 12] varied from 10 to 15 days, likely indicative of a much larger physiologic insult than even large extremity STS resections. In addition, postoperative mobilization in our patient population is less likely to be limited by nausea, vomiting, ileus, and atelectasis thus possibly allowing for earlier discharges and less need for rehab or nursing facility placement.

We recommend performing further prospective clinical studies with larger sample sizes analyzing STS outcomes and correlating them with skeletal muscle mass and nutritional markers such as albumin and C-reactive protein. In addition, comparison of the molecular signaling pathways between sarcoma and carcinoma cachexia is needed to elucidate the likely differences between the two.

## 5. Conclusions

Sarcopenia, as manifested by decreased height-adjusted TPA, does not impact overall survival, hospital LOS, or wound complications including wound infection in patients with extremity STS. Biological, molecular, and pathological differences between patients with soft-tissue sarcomas and carcinomas may explain the differential effects of sarcopenia on patient outcomes.

## Figures and Tables

**Table 1:** Demographic and clinical statistics.

		Range
Median age (years)	59	(42–70)
Median height (meters)	1.72	(1.6–1.8)

		Percentage

Gender	Male (*N* = 69)	50%
	Female (*N* = 68)	50%
Body mass index (BMI)	BMI < 30 (*N* = 86)	63%
	BMI 30–35 (*N* = 30)	22%
	BMI 35–40 (*N* = 13)	9%
	BMI 40–45 (*N* = 4)	3%
	BMI > 45 (*N* = 4)	3%
Surgery	Wide local excision (*N* = 126)	92%
	Amputation (*N* = 11)	8%
Surgical margins	Negative (*R*_0_) (*N* = 121)	88%
	Positive (*R*_1_) (*N* = 16)	12%

		Range

Median follow-up (years)	4.1	(1.7–5.6)
Median tumor size (cm)	9.0	(5.0–15.0)
Median time to death (months)	69	(40–89)

		Percentage

Tumor depth (relative to investing fascia)	Superficial (*N* = 28)	20%
	Deep (*N* = 109)	80%
Tumor grade	Low (*N* = 25)	18%
	Intermediate (*N* = 15)	11%
	High (*N* = 97)	71%
Histologic sarcoma type	Fibrosarcoma (*N* = 5)	4%
	Leiomyosarcoma (*N* = 16)	12%
	Liposarcoma (*N* = 30)	22%
	Malignant peripheral nerve sheath tumor (*N* = 3)	2%
	Undifferentiated pleiomorphic sarcoma (*N* = 57)	42%
	Synovial sarcoma (*N* = 7)	5%
	Vascular sarcoma (*N* = 5)	4%
	Rhabdomyosarcoma (*N* = 1)	1%
	Others (*N* = 13)	9%
AJCC stage at presentation	I (*N* = 34)	25%
	II (*N* = 27)	20%
	III (*N* = 33)	24%
	IV (*N* = 42)	31%
Preoperative radiation	Yes (*N* = 32)	23%
	No (*N* = 105)	77%
Postoperative radiation	Yes (*N* = 84)	61%
	No (*N* = 53)	39%
Chemotherapy	Yes (*N* = 28)	21%
	No (*N* = 106)	79%
Survival status at last follow-up	Alive (*N* = 94)	69%
	Died of disease (*N* = 23)	17%
	Died of other causes (*N* = 10)	7%
	Uncertain cause of death (*N* = 5)	4%

**Table 2:** Outcome variables by gender.

	Male (*N* = 69)	Female (*N* = 68)	*p* value
Average height-adjusted TPA (cm^2^/m^2^)	7.3	5.2	<0.001^2^
Median height-adjusted TPA (range, cm^2^/m^2^)	7.3 (6.1–8.2)	4.9 (4.4–5.9)	
Wound infection			0.12^1^
Yes (*N* = 23)	15	8	
No (*N* = 114)	54	60	
Wound complications			0.064^1^
Yes (*N* = 38)	24	14	
No (*N* = 99)	45	54	
Wound dehiscence			0.14^1^
Yes (*N* = 28)	15	13	
No (*N* = 10)	8	2	
Wound necrosis			0.8^1^
Yes (*N* = 11)	7	4	
No (*N* = 27)	16	11	
Average length of hospital stay (days)	3.9	4.3	0.7^2^
Median length of hospital stay (range in days)	3.0 (3.0–4.2)	3.0 (3.0–6.0)	
Postsurgical disposition			0.8^1^
Home (*N* = 116)	57	59	
Home with home health physical therapy (*N* = 5)	4	1	
Skilled nursing facility (*N* = 5)	2	3	
Inpatient rehab (*N* = 6)	3	3	
Transfer to another hospital (*N* = 3)	2	1	
Unknown disposition (*N* = 2)	1	1	

1 = Pearson test, 2 = Wilcoxon test.

**Table 3:** Results of Cox proportional hazard model for height-adjusted total psoas area (TPA) and overall survival.

Variable	*X* ^2^	d.f. (degrees of freedom)	*p* value
Height-adjusted TPA	0.105	1	0.746
Height-adjusted TPA controlling for gender	0.137	1	0.712
Patient age	5.403	1	0.02
Tumor size	6.796	1	0.009
Tumor grade	10.11	1	0.001
Tumor depth	0.920	1	0.337
*R*_1_ resection margin (positive margin)	0.645	1	0.422

**Table 4:** Linear analysis of height-adjusted total psoas area (TPA) and hospital LOS, postoperative infection, and wound complications.

TPA category	Variable	*X* ^2^	d.f. (degrees of freedom)	*p* value
Height-adjusted TPA	Hospital LOS^*∗*^		4	0.66
	Wound infection	1.16	2	0.56
	Wound complications	3.92	2	0.14

Height-adjusted TPA controlling for gender	Hospital LOS^*∗*^		2	0.34
	Wound infection	1.02	1	0.31
	Wound complications	1.28	1	0.26

^*∗*^Analysis of variance test performed instead of chi-squared analysis due to LOS being a noncategorical variable.
